# Integration of ^1^H NMR and UPLC-Q-TOF/MS for a Comprehensive Urinary Metabonomics Study on a Rat Model of Depression Induced by Chronic Unpredictable Mild Stress

**DOI:** 10.1371/journal.pone.0063624

**Published:** 2013-05-17

**Authors:** Hong-mei Jia, Yu-fei Feng, Yue-tao Liu, Xing Chang, Lin Chen, Hong-wu Zhang, Gang Ding, Zhong-mei Zou

**Affiliations:** 1 Institute of Medicinal Plant Development, Chinese Academy of Medical Sciences and Peking Union Medical College, Beijing, PR China; 2 Department of Pharmacy, Beijing Hospital, Ministry of Public Health, Beijing, PR China; Moffitt Cancer Center, United States of America

## Abstract

Depression is a type of complex psychiatric disorder with long-term, recurrent bouts, and its etiology remains largely unknown. Here, an integrated approach utilizing ^1^H NMR and UPLC-Q-TOF/MS together was firstly used for a comprehensive urinary metabonomics study on chronic unpredictable mild stress (CUMS) treated rats. More than twenty-nine metabolic pathways were disturbed after CUMS treatment and thirty-six potential biomarkers were identified by using two complementary analytical technologies. Among the identified biomarkers, nineteen (**10, 11,** **16, 17, 21–25,** and **27–36**) were firstly reported as potential biomarkers of CUMS-induced depression. Obviously, this paper presented a comprehensive map of the metabolic pathways perturbed by CUMS and expanded on the multitude of potential biomarkers that have been previously reported in the CUMS model. Four metabolic pathways, including valine, leucine and isoleucine biosynthesis; phenylalanine, tyrosine and tryptophan biosynthesis; tryptophan metabolism; synthesis and degradation of ketone bodies had the deepest influence in the pathophysiologic process of depression. Fifteen potential biomarkers (**1**–**2**, **4**–**6**, **15**, **18**, **20**–**23**, **27**, **32**, **35**–**36**) involved in the above four metabolic pathways might become the screening criteria in clinical diagnosis and predict the development of depression. Moreover, the results of Western blot analysis of aromatic L-amino acid decarboxylase (DDC) and indoleamine 2, 3-dioxygenase (IDO) in the hippocampus of CUMS-treated rats indicated that depletion of 5-HT and tryptophan, production of 5-MT and altered expression of DDC and IDO together played a key role in the initiation and progression of depression. In addition, none of the potential biomarkers were detected by NMR and LC-MS simultaneously which indicated the complementary of the two kinds of detection technologies. Therefore, the integration of ^1^H NMR and UPLC-Q-TOF/MS in metabonomics study provided an approach to identify the comprehensive potential depression-related biomarkers and helpful in further understanding the underlying molecular mechanisms of depression through the disturbance of metabolic pathways.

## Introduction

Metabonomics, with its impressive and ever-increasing coverage of endogenous compounds, has been successfully employed in many areas, including the pharmaceutical industry, food safety and exploration of pathogenesis [1]. More importantly, metabonomics analysis can offer an unbiased view of changes in metabolism, cover entire metabolic pathways to characterize pathological states, and provide diagnostic information [2].

However, metabonomics analysis still faces many challenges and these challenges arise from the complexity of the metabolite composition and limitations of a given analytical method (NMR, GC-MS, LC-MS, and CE-MS). On one hand, the metabolites of biofuilds or tissue samples are diverse in their physical and chemical properties and occur over wide concentration ranges [3]. On the other hand, neither of two existing main stream methods (i.e., NMR and LC-MS) alone can fully meet such requirements due to their intrinsic limitations in either detection or quantification. Therefore, the integration of multiple analytical platforms would make up for the deficiencies in different technologies and provide greater scientific power to metabolic disturbances [4].

Depression is a type of long-term, complex psychiatric disorder characterized by repeated outbreaks [5]. Current clinical diagnosis of depression relies on a trained clinician making a decision based upon patient medical history and presentation of symptoms. The etiology of depression is not yet fully understood and diagnosis of depression remains subjective based on descriptive symptoms by depression patients, due to the lack of objective index in clinic [6]. Discovery of depression related biomarkers with the help of non-invasive and unbiased approaches are required for the prediction and diagnosis.

Chronic unpredictable mild stress (CUMS) can produce a series of abnormal behavioral and physiological responses which are similar to the depressive symptoms of human, and is often used as an animal model to study the pathogenesis of depression [7]. In previous studies, the studies on the metabolic disorders of urine [8]–[14], plasma [15]–[19] and brain tissues [20] from the CUMS-treated rats have been reported with the aims to elaborate the biochemical responses to stress and evaluate the antidepressant effects of diverse drugs. The most used analytical platforms in the metabonomics study of CUMS-induced depressive rats involved in depression are GC-MS [13], [15], [16], [20], NMR [11], [12], [17], [18], or LC-MS [8], [9], [10], [14]. Up to now, only one metabonomic study on the plasma of CUMS-treated rats was conducted by using two complementary analytical technologies [19]. Here, a comprehensive urinary metabonomics study on a rat model of depression induced by CUMS was explored using a combination of ^1^H NMR and UPLC-Q-TOF/MS with the aims to achieve the most comprehensive metabolome coverage and provide a more in-depth understanding of the pathophysiological processes of depression (Figure S1).

## Materials and Methods

### Chemicals and Reagents

HPLC-grade acetonitrile was purchased from Merck (Darmstadt, Germany). The water used for UPLC was purified by a Milli-Q system (Millipore, France). Formic acid (HPLC grade) was obtained from Tedia (Fairfield, USA), and Leucine-enkephalin was obtained from Sigma Aldrich (St. Louis, MO, USA). Na_2_HPO_4_⋅12H_2_O and NaH_2_PO_4_⋅2H_2_O were all purchased as analytical grade from Guoyao Chemical Co. Ltd.(Shanghai, China) and used without further treatments. Deuterium oxide (D_2_O, 99.9%, contains 0.05 wt% sodium 3-trimethylsilyl [2, 2, 3, 4-^2^H_4_] propionate (TSP) was purchased from Cambridge Isotope Laboratories, Inc. (MA, USA). All other chemicals used were of analytical grade. Antibodies against DDC (aromatic L-amino acid decarboxylase) and IDO (indoleamine 2, 3-dioxy-genase) were purchased from Abcam (Cambridge, Britain).

### Animals and Treatments

Sixteen healthy, adult, male Wistar rats, weighing 200±20 g each, were purchased from the Institute of Laboratory Animal Science, CAMS & PUMC (Beijing, China). The rats were housed individually in cages for one week to adapt to the environment under controlled conditions of 12 h light-12 h dark cycles (lights on from 6∶00 a.m.–6∶00 p.m.), 10% relative humidity and temperature (20±3°C) with commercial diet and water available *ad libium*. All experimental procedures were approved by the Ethics Committee of the Institute of Medicinal Plant Development, CAMS & PUMC.

The animals were randomly divided into two groups. Untreated rats served as the naïve group, and the CUMS-treated rats were subjected to a series of variable stimuli as previously described [8]; the stimuli included the following: (1) immobilization for 5 h, (2) swimming in 15°C water for 5 min, (3) withholding food for 48 h, (4) swimming in 45°C water for 5 min, (5) withholding water for 48 h, (6) electric shock to pelma (electric current for 1 mA, 2 s per shock, 2 shocks per minute), (7) noise stimulus at 11 dB, (8) stroboflash-2 flashes per second for 4 h. During a period of 28 d, one of the stressors was chosen randomly and performed such that the rats did not expect the stimulus. Every stressor was used 2–3 times in total.

### Sample Collection

All rats were housed in metabolic cages (1 per cage) so that the 24 h urine sample could be collected in collection bottles containing NaN_3_ (0.05% wt/vol) on the 28th day. The urine samples were centrifuged (5000 *rpm* for 10 min, 4°C), and the supernatants were stored at −80°C until NMR and LC-MS analysis.

At the end of 28 days, the rats were all sacrificed. The hippocampus was immediately removed at 4°C and rinsed with saline, blotted on filter paper, weighed and stored at −80°C for Western blot analysis.

### ^1^H NMR Analysis

#### Sample preparation

An aliquot of 400 µL urine was thawed at room temperature and mixed with 200 µL of phosphate buffer [0.2 M Na_2_HPO_4_ and 0.2 M NaH_2_PO_4_ in D_2_O containing 0.05% wt/vol 3-trimethylsilyl-(2,2,3,3-^2^H_4_)-1-propionate (TSP); pH 7.4]. Phosphate buffer minimized chemical shift variation because of different pH in urine samples, with D_2_O as a field lock and TSP as a chemical shift reference. The mixture was centrifuged (13,000 *rpm*, 15 min, 4°C), and the supernatant (550 µL) of each sample was then transferred into a 5-mm o.d. NMR tube.

#### NMR detection experiment parameters

All ^1^H NMR spectra were recorded at 300 K on a Bruker AV III 600 spectrometer (Bruker Biospin, Germany) equipped with an inverse 5-mm Bruker probe operating at 600.13 MHz ^1^H frequency. ^1^H NMR spectra were acquired using water-suppressed NOSEYGPPR1D (RD-90-t-90-t_m_-90-ACQ); water signal suppression was achieved with weak irradiation on the water peak during the recycling delay (RD = 4.0 s) and mixing time (t_m_ = 0.10 s). The 90° pulse length was adjusted to ∼10 µs. A total of 128 transients were collected into 96 K data points over a spectral width of 20 ppm with an acquisition time of 3.07 s.

#### Data processing

Prior to Fourier transformation, the FIDs for one-dimensional data were multiplied by an exponential function equivalent to a line-broadening factor of 0.5 Hz and zero-filled to 128 K. All NMR spectra were then corrected for phase and baseline distortions using Topspin software (v2.1, Bruker-Biospin, Germany). ^1^H NMR chemical shifts in the spectra were referenced to TSP at *δ* 0.00. The spectra were divided, and the signal integral was computed in 0.004 ppm intervals across the region *δ* 0.50–9.50 using the AMIX software package (v3.9.2, Bruker-Biospin, Germany). The region *δ* 4.67–5.10 was removed to avoid the effect of residual water saturation, leaving 1875 variables.

### UPLC-Q-TOF/MS Analysis

#### Sample preparation

All urine samples were thawed at room temperature before analysis and centrifuged at 13,000 *rpm* for 10 min at 4°C. The supernatant was diluted at a ratio of 1∶1 with water and an aliquot of 5 µL was injected for UPLC analysis after filtration through a 0.22**µM membrane filter.

#### Method development and validation

The urine samples were analyzed on a Waters Acquity™ Ultra Performance LC system (Waters Corporation, Milford, MA, USA) equipped with a BEH C18 column (100 mm×2.1 mm, 1.7 µm). The mobile phase was composed of water (A) and acetonitrile (B), each containing 0.1% formic acid. The following solvent gradient system was used: 1% B from 0 to 1 min, 1–32% B from 1 to 9 min, 32–99% B from 9 min to 11 min, and 99% B from 12–15 min. The flow rate was 0.45 mL min^−1^. All the samples were kept at 4°C during the analysis. The mass spectrometric data were collected using a Q-TOF analyzer in a SYNAPT HDMS system (Waters Corporation, Milford, MA, USA) in both positive and negative ion modes. The parameters were set as previously described [8].The source temperature was set at 120°C with a cone gas flow of 50 LH^−1^, a desolvation gas temperature of 450°C and a desolvation gas flow of 800 LH^−1^. For the positive and negative ion modes, the capillary voltage was set to 3.0 kV and 2.5 kV, respectively, and the cone voltage was set to 35 V. Centroid data were collected from *m/z* 50 to 1200 with a scan time of 0.3 s and an interscan delay of 0.02 s over a 15 min analysis time. Leucine-enkephalin was used as the lock mass (*m/z* 556.2771 in positive mode and *m/z* 554.2615 in negative mode) at a concentration of 0.5 µgmL^−1^ with a flow rate of 80 µLmin^−1^. The lock spray frequency was set at 20 s.

To ensure the reproducibility of the developed method, we examined its precision and repeatability. We obtained a 100 µL urine sample from each animal, mixed and processed it as the sample preparation, and then used the supernatant as the QC sample. The extracted ion chromatographic peaks of ten ions were selected for method validation. The repeatability of the method was evaluated using 6 replicates of the QC sample. The precision of the injection was assessed using 6 replicated analyses of the same urine sample. The relative standard deviations (R.S.D %) of the retention time and *m/z* were listed in Table S2.

#### Data processing

The raw data were analyzed using the MarkerLynx Applications Manager version 4.1 (Waters, Manchester, U.K.), which allowed for deconvolution, alignment and data reduction to provide a list of retention time and mass pairs with corresponding intensities for all of the detected peaks from each data file in the data set. The main parameters were set as follows: retention time (RT) range 0.5–15.0 min, mass range 50–1200 amu, mass tolerance 0.02, minimum intensity 1%, mass window 0.05, retention time window 0.20, and noise elimination level 6.

### Protein Extraction and Western Blot Analysis

Protein was extracted from 50 mg whole, frozen rat hippocampus tissue with 500 µL RIPA lysis buffer (50 mM Tris-HCl pH 8.0, 150 mM NaCl, 1% NP-40, 1% deoxycholic acid sodium, 0.1% SDS, 1 mM PMSF, Roche Complete protease inhibitor cocktail tablets and phosphatase inhibitor cocktail tablets) in a 2 mL microcentrifuge tube. The tissue was cut into small pieces and homogenized the pieces with a homogenizer (Fluko) at 15,000 *rpm* for 30 s. After homogenization, the samples were incubated in ice for 20 min and centrifuged for 20 min at 13,000 *rpm* at 4°C. The supernatant was collected, and the protein concentration was determined by BCA.

For Western blot analysis, we used equal amounts of proteins. Briefly, 20 µg of tissue lysate were subjected to electrophoresis on 12% SDS-PAGE gels, and the separated proteins were electrophoretically transferred onto nitrocellulose membranes. The membranes were rinsed twice with TBS and Tween 20 (TBST)/Tris-buffered saline, and then incubated with a blocking buffer (5% BSA/TBST) for 30 min at room temperature. Overnight incubation of the membranes with primary antibodies [anti-aromatic L-amino acid decarboxylase (DDC), 1∶2000 dilutions, and anti-indoleamine 2, 3-dioxy-genase (IDO), 1∶1500 dilutions] was performed at 4°C, followed by six 3-min washes with TBST. The membranes were incubated with secondary antibodies at room temperature for 40 min and washed six times with TBST; the antibody-bound proteins were detected using enhanced chemiluminescence reagents (Millipore), according to the manufacturer’s protocol. To calculate the fold change, the density of the protein bands was determined using the Image Quant TL software provided by GE. After normalization to *β*-actin, the control sample was assigned an arbitrary value of 1.

### Data Analysis

To diminish the deviation in data analysis from individual variance of urine samples, data were normalized by a creatinine calibration method, i.e. the metabolite intensity was divided by the creatinine concentration each sample. Then according to the 80% rule [21], [22], only variables having more than 80% nonzero measurement values were kept in the peak list.

The NMR spectral data and the resulting UPLC-MS data were introduced to SIMCA-P software package (v12.0, Umetric, Umeå, Sweden) for principal component analysis (PCA) and orthogonal partial least squares discriminate analysis (OPLS-DA). Imported data were mean-centered and pareto-scaled prior to multivariate analysis. Mean centering calculates the average spectrum of the data set and subtracts that average from each spectrum, aiming to focus o the fluctuating part of data insteps of the original value. Pareto (Par) scaling was used in all the models to avoid chemical noise. PCA and OPLS-DA were employed to process the acquired NMR and MS data. PCA was performed to discern the natural separation between different stages of samples by visual inspection of score plots (Figure S2). In the OPLS-DA model, samples from different groups were classified, and the results were visualized in the form of score plots to show the group clusters. Potential biomarkers were selected according to Variable importance in the Project (VIP) value, the loading plot and the S-plot.

A two-tailed Student’s *t*-test was performed using the Statistical Package for Social Science program (SPSS 16.0, SPSS, Chicago, IL, USA). The significance threshold was set at *p*<0.05 for this test.

## Results

### Ethological changes in the CUMS-treated Rats

The ethologies of the CUMS-treated rats were evaluated by body weight, the number of horizontal movement and the sucrose preference [23]. After 28 days of chronic stress exposure, the ethology of CUMS-treated rats were significantly changed compared with the naïve group (Figure S3) which indicated that CUMS treatment led to a series of impairments in rats similar to the symptoms observed in depressed patients, such as lack of acute activation, weakening of the functions of the digestion system and anhedonia.

### Urine Metabolic Profile based on ^1^H NMR Technology

^1^H NMR was used to obtain urinary metabolic profiles of CUMS-treated rats and naïve rats at day 28. Typical ^1^H NMR spectra of urine samples from CUMS-treated rats and naïve rats were shown in supporting information (Figure S4) and the metabolites were identified based on their characteristic chemical shifts and multiplicities according to the literatures [24]–[27] and the Human Metabonome Database (http://www.hmdb.ca/).

**Figure 1:**
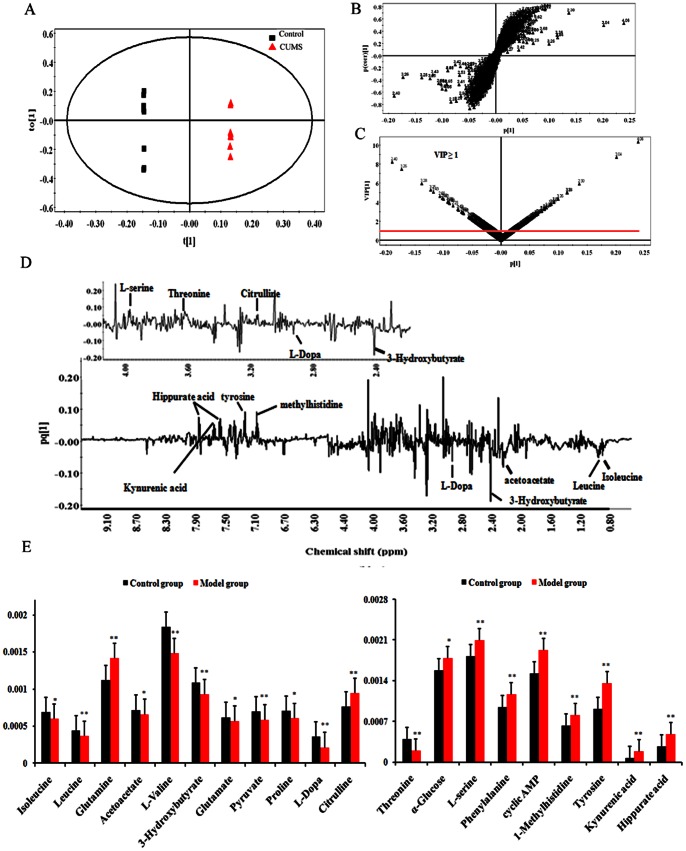
OPLS-DA-based ^1^H NMR data of urine samples obtained from CUMS and naïve rats. A, score plots; B, loading plots; C, VIP plots; D, loading line plots; E, mean peak intensity of the 20 representative metabolites between the CUMS group and naïve group (*p<0.05, **p<0.01).

PCA (Figure S2) was firstly carried out to investigate whether two groups can be separated and to find out their metabolic distinction. Then, OPLS-DA, a supervised multivariable statistical method to sharpen an already established separation between groups of observations plotted in PCA was performed (Figure 1A). The results indicated that the metabolic profile of rat in CUMS-treated group deviated from the naïve group, suggesting that significant biochemical changes were induced by CUMS. The S-plot of OPLS-DA (Figure 1B) indicated the variables responsible for the differentiation, and the variable importance for projection (VIP) value signifies the influence of the metabolites on the classification. Variables far from the origin in the S-plot with VIP values ≥1 (Figure 1C) contributed significantly to the clustering and were considered as potential biomarkers. The directions of the resonance signals in the loading line plot (Figure 1D) indicate the variation tendency of the potential biomarkers, in which the upward peaks represent the growth content and the downward peaks represent the decline content of the corresponding metabolite, respectively.

The significant variables detected by NMR were summarized in Table 1 and Figure 1E. Among them, isoleucine (**1**), leucine (**2**), acetoacetate (**4**), valine (**5**), 3-hydroxybutyrate (**6**), glutamate (**7**), pyruvate (**8**), proline (**9**), L-dopa (**10**) and threonine (1**2**) decreased in the urine samples from the CUMS-treated rats compared with those from the naïve rats. Conversely, glutamine (**3**), citrulline (**11**), *α*-glucose (1**3**), L-serine (1**4**), phenylalanine (**15**), cyclic AMP (**16**), 1-methylhistidine (**17**), tyrosine (**18**), hippurate acid (**19**), and kynurenic acid (**20**) increased.

**Table 1:** The potential biomarkers detected by ^1^H NMR of CUMS-induced depression and their variation tendency.

NO.	Metabolite	Chemical shift	VIP	Model	Metabolic Pathway
1	isoleucine	0.94	2.27	↓**	Valine, Leucine and Isoleucine Degredation
2	leucine	0.95,0.96	2.11	↓**	Valine, Leucine and Isoleucine Degredation
3	glutamine	2.08	1.12	↑**	Glutamate Metabolism; Purine Metabolism; Urea Cycle
4	acetoacetate	2.22	2.06	↓**	Ketone Body Metabolism
5	valine	2.24	3.19	↓**	Valine, Leucine and Isoleucine Degradation
6	3-hydroxybutyrate	2.31,2.33,2.38	2.04	↓**	Ketone Body Metabolism
7	glutamate	2.36	1.98	↓**	Alanine Metabolism; Cysteine Metabolism
8	pyruvate	2.48	1.99	↓**	Ketone Body Metabolism
9	proline	2.66	1.60	↓**	Arginine and Proline Metabolism
10	L-dopa	2.96	1.75	↓**	Tyrosine Metabolism
11	citrulline	3.15	2.41	↑**	Aspartate Metabolism
12	threonine	3.6	2.50	↓**	Glycine and Serine Metabolism
13	*α*-glucose	3.86	3.06	↑**	Galactose Metabolism
14	L-serine	3.96	3.80	↑**	Glycine and Serive Metabolism
15	phenylalanine	3.97,7.32	3.43	↑**	Phenylalanine and Tyrosine Metabolism
16	cyclic AMP	4.52	1.59	↑**	Purine Metabolism
17	1-methylhistidine	7.05	2.49	↑**	Histidine Metabolism
18	tyrosine	7.22	3.98	↑**	Tyrosine Metabolism
19	hippurate acid	7.55,7.57,7.84	3.08	↑**	Phenylalanine Metabolism
20	kynurenic acid	7.64	2.29	↑**	Phenylalanine Metabolism

Variations metabolites in CUMS-treated rats compared to naïve group. “↑”, increase in signal; “↓”, decrease in signal, *p<0.05, **p<0.01.

### Urine Metabolic Profile Based on UPLC-Q-TOF/MS Technology

**Figure 2:**
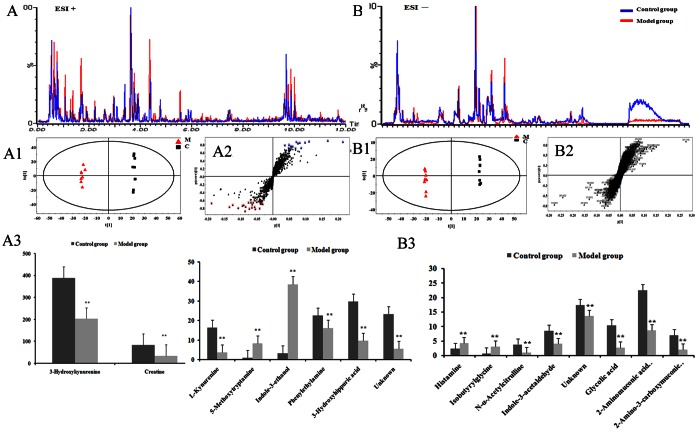
OPLS-DA based on positive and negative UPLC-Q-TOF/MS data of urine samples obtained from CUMS and naïve rats. A, B: Base peak chromatography; A1, B1: scores plots; A2, B2: S-plots; A3, B3: mean peak area of the 16 representative metabolites between the CUMS group and naïve group (*p<0.05, **p<0.01).

Metabolic profiles of urine samples were also performed using UPLC-Q-TOF/MS in the positive and negative ion scan modes (Figure S5). The representative base peak intensity (BPI) chromatograms of urine samples from the naïve and CUMS-treated rats were shown in Figure 2A and 2B.

The score plots of OPLS-DA (Figure 2A1 and 2B1) obtained from the UPLC-Q-TOF/MS data showed that the CUMS model group and naïve group can be successfully differentiated in both positive ion and negative ion modes by the first principal component with statistical significance (*p*<0.05). The urine metabolic profiles of CUMS-treated rats deviated from that of the naïve, suggesting that significant biochemical changes were induced by CUMS. Loading plots (Figure 2A2 and 2B2) indicated that sixteen metabolites with high variable importance (VIP≥1) (Table 2) were responsible for the discrimination in the score plots. The structures of those metabolites were identified by analyzing their accurate molecular weights and MS/MS spectra [28]–[31]. Databases, such as HMDB, METLIN, MassBank and KEGG were used for confirmation.

**Table 2:** The potential biomarkers detected by UPLC-Q-TOF/MS of CUMS-induced depression and their variation tendency.

ESI+						ESI-					
NO.	Metabolite	RT	M/Z	Model	VIP	NO.	Metabolite	RT	M/Z	Model	VIP
21	L-kynurenine	0.64	209.2137	↓**	3.50	29	histamine	0.7	110.1449	↑**	1.60
22	5-methoxytryptamine	1.26	175.0822	↑**	2.53	30	isobutyrylglycine	1.15	144.1583	↑**	1.41
23	indole-3-ethanol	3.37	162.2083	↑**	4.55	31	N-a-Acetylcitrulline	3.77	216.108	↓**	1.43
24	phenylethylamine	4.32	122.1795	↓**	4.45	32	indole-3-acetaldehyde	4.59	158.1023	↓**	1.98
25	3-hydroxyhippuric acid	4.45	196.1710	↓**	3.92	33	Unidentified	4.84	203.1289	↓**	2.01
26	Creatine	4.65	132.0951	↓**	4.05	34	glycolic acid	6.16	75.0619	↓**	2.18
27	3-hydroxykynurenine	4.86	225.1091	↓**	10.62	35	2-aminomuconic acid semialdehyde	9.29	140.1169	↓**	4.25
28	Unidentified	5.48	297.1664	↓**	3.45	36	2-amino-3-carboxymuconic acid semialdehyde	9.69	184.1278	↓**	2.61

Variations metabolites in CUMS-treated rats compared to naïve group. “↑”, increase in signal; “↓”, decrease in signal, *p<0.05, **p<0.01.

Kynurenine (**21**), phenylethylamine (**24**), 3-hydroxyhippuric acid (**25**), creatine (**26**), 3-hydroxykynurenine (**27**), N-α-acetylcitrulline (**31**), indole-3-acetaldehyde (**32**), glycolic acid (**34**), 2-aminomuconic acid semialdehyde (**35**), and 2-amino-3-carboxymuconic acid semialdehyde (**36**) in the CUMS-treated rats decreased significantly, whereas 5-methoxytryptamine (**22**), indole-3-ethanol (**23**), histamine (**29**) and isobutyrylglycine (**30**) increased significantly (Figure 2A3 and 2B3).

### Perturbed Metabolic Pathways in Response to CUMS

Both the NMR-based and LC-MS-based metabonomics studies indicated that the metabolic profile of the CUMS-treated rats deviated from that of the naïve, suggesting that urinary biochemical changes occurred in the CUMS-treated rats. Based on the identified potential biomarkers using ^1^H NMR and UPLC-Q-TOF/MS, a comprehensive metabolic network of CUMS induced depression was mapped on MetaboAnalyst 2.0 [http://www.metaboanalyst.ca/MetaboAnalyst/] [32]. More than twenty-nine metabolic pathways were disturbed after CUMS treatment (Table S3, Figure S6). The impact value of pathways calculated from pathway topology analysis was applied to evaluate the impact of the pathways on the development of depression.

**Figure 3:**
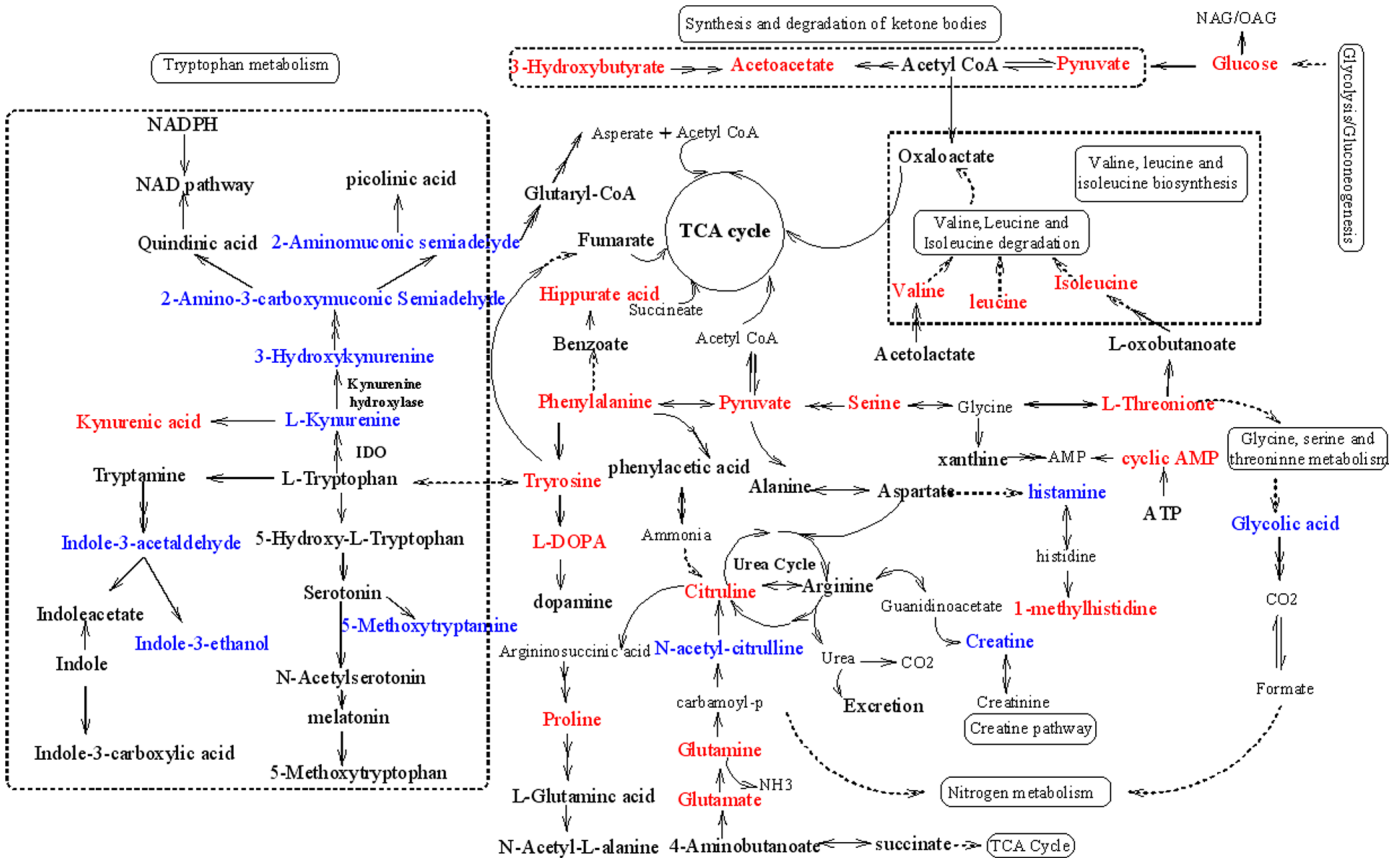
An overview of the metabolic pathways related to CUMS-induced depression. Red-labeled metabolites were detected by ^1^H-NMR, and blue-labeled metabolites were detected by UPLC-Q-TOF/MS.

Here,those pathways with the impact value>0.5 were considered as the most relevant pathways involved in CUMS induced depression. There are four metabolic pathways (Table S3), including valine, leucine and isoleucine biosynthesis; phenylalanine, tyrosine and tryptophan biosynthesis; tryptophan metabolism; and the synthesis and degradation of ketone bodies, which were the most influenced metabolic pathways associated with CUMS-induced depression (Figure 3).

#### Valine, leucine and isoleucine biosynthesis

Isoleucine (**1**), leucine (**2**) and valine (**5**) are proteinogenic amino acids with aliphatic side-chains and are called branched-chain amino acids (BCAAs). BCAAs can be quickly transported across the blood-brain barrier as major amino group donors for the synthesis of glutamate in the brain [33]. Glutamate, which is an important neurotransmitter, plays a key role in long-term potentiation, learning and memory. Additionally, decreased synthesis of glutamate results in depression-like behaviors [34]. Isoleucine (**1**) and leucine (**2**) [18], [20] were previously detected in brain tissue and plasma sample, and valine (**5**) [13] in urine sample of the CUMS-treated rats. Here,we found that the concentrations of isoleucine (**1**), leucine (**2**) and valine (**5**) were significantly decreased in the urine samples of the CUMS-treated rats. Those findings indicated that CUMS treatment inhibited the biosynthesis of these branched-chain amino acids and blocked glutamate synthesis in CUMS-treated rats.

#### Phenylalanine, tyrosine and tryptophan biosynthesis

Phenylalanine (**15**), tyrosine (**18**) and tryptophan are the materials required for the biosynthesis of the monoamine neurotransmitter and play an important role in the pathogenesis of depression [35]. Abnormalities in the metabolisms of tryptophan and tyrosine were found in depressed patients by plasma biochemical analysis [36]. Here, the increased phenylalanine (**15**) and tyrosine (**18**) were detected in the urine of CUMS-treated rats, which was in agreement with the previous reports [9], [14].

#### Tryptophan metabolism

**Figure 4:**
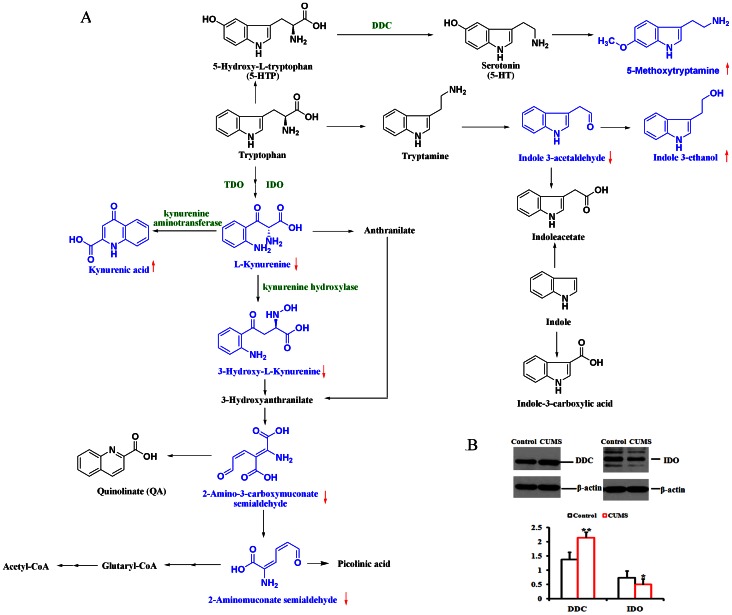
The map of Tryptophan metabolism and the results of Western blot analysis of DDC and IDO in the rat’s hippocampus. (A) The serotonin synthesis (5-HT) pathway and kynurenine pathway in tryptophan metabolism. The structure in blue indicates the compounds that were significantly changed in the CUMS group. (B) The results of Western blot analysis of DDC and IDO in the rat’s hippocampus. Each measure was performed with three replicates and was expressed as the mean ± SEM (*p<0.05, **p<0.01).

Tryptophan (TRP), the least abundant essential amino acid, is involved in both the 5-HT metabolism and kynurenine pathways (KP) (Figure 4A). The synthesis of 5-HT in the brain is highly dependent on the bio-availability of TRP. The first and rate-limiting step in the biosynthesis of 5-HT is the hydroxylation of tryptophan to 5-hydroxytryptophan (5-HTP). And then, 5-HT was further metabolized into N-methylserotonin, melatonin, and 5-methoxytryptamine (5-MT). In present study, the level of 5-MT (**22**) was increased significantly in the urine samples of CUMS-treated rats, which indicated that CUMS would lead to the depletion of 5-HT and result in the symptoms of depression.

The kynurenine pathway of tryptophan metabolism converts the amino acid tryptophan into a number of biologically active metabolites [37]. The first step of this pathway, conversion of tryptophan to L-kynurenine (KYN), is rate-limiting. KYN is further metabolized into 3-hydroxykynurenine (3-HK) and quinolinic acid (QUIN), which may induce neuronal damage through oxidative stress and overstimulation of the N-methyl-D-aspartate (NMDA) receptor. KYN is further metabolized into 3-hydroxykynurenine (3-HK) and 3-hydroxyanthranilic acid (3-HAA). Present study detected abnormality of five products of kynurenine pathway (KP), including the increased level of kynurenic acid (**20**) and the decreased levels of L-kynurenine (**21**), 3-hydroxykynurenine (**27**), 2-aminomuconic acid semialdehyde (**35**), and 2-amino-3-carboxymuconic acid semialdehyde (**36**) in the urine of CUMS-treated rats (Figure 3).

Another two metabolites involved in the tryptophan metabolism, indole-3-ethanol (**23**) and indole-3-acetaldehyde (**32**), were also detected in the present study. A total of eight metabolites (**20**–**23**, **27**, **32**, **35**, and **36**) involved in tryptophan metabolism were identified as potential biomarkers of CUMS-induced depression. Apart from kynurenic acid (20) [8], [14], the others were firstly identified as potential biomarkers associated to CUMS-induced depression.

#### Synthesis and degradation of ketone bodies

Glucose and ketone bodies are the only energy providers for the brain because of the existence of the blood-brain barrier. Ketone bodies, the intermediate of fatty acids metabolism, provide a protective effect for the central nervous system [38]. Two important ketone bodies, acetoacetate (**4**) and 3-hydroxybutyric acid (**6**) detected by NMR, were decreased in the urine samples of the CUMS-treated rats, indicated that ketone bodies metabolism played a role in the CUMS-induced depression.

### The Expression of Aromatic L-amino Acid Decarboxylase and Indoleamine 2, 3-dioxy-genase

Aromatic L-amino acid decarboxylase (DDC, EC 4.1.1.28) [39] catalyzes several different decarboxylation reactions, including L-dopa to dopamine, 5-HTP to 5-HT and tryptophan to tryptamine. The corresponding enzyme-catalyzed reaction substrates and products are critical neurotransmitters in the central nervous system (CNS) and play important roles in the pathogenesis of depression. Here, we observed the up-regulation of DDC in the hippocampus of rats after CUMS treatment (Figure 4B). Consequently, L-DOPA, 5-HTP and tryptophan were decomposed to dopamine, 5-HT and tryptamine, respectively, by activation of DDC. Indeed, we observed a decreased level of L-dopa (**10**) in our metabonomics study (Table 1). Although the excretion of 5-HT was not significantly increased in the urine of CUMS-treated rats, the level of 5-MT was significantly increased compared with naïve rats.

Indoleamine 2, 3-dioxy-genase (IDO, EC: 1.13.11.52), the first enzyme in the KYN pathway, catalyzes the conversion of tryptophan to kynurenine [40]. The level of IDO in the hippocampus of the CUMS-treated rat was decreased (Figure 4B) compared with the level in the control rat. Consequently, decreased levels of L-kynurenine (**21**) and its metabolites, including 3-hydroxykynurenine (**27**), 2-aminomuconic acid semialdehyde (**35**) and 2-amino-3-carboxymuconic acid semialdehyde (**36**), were detected in our metabonomics study. The low levels of these metabolites reflected the depletion of tryptophan because the activation of DDC degrades TRP and 5-HTP to offset the consumption of 5-HT for the synthesis of 5-MT.

## Discussion

Metabonomics focus on the systematic study of the full complement of metabolites in a range of biofluids including urine, plasma, serum, cerebrospinal fluid (CSF), synovial fluid, semen, and tissue homogenates [1]. Among them, urine is a key biological matrix in metabolic profiling studies, as its collection is noninvasive and simple, and urine samples are less likely to be volume-limited [2]. Furthermore, as urine is not under homeostatic regulation, being a waste product, it can reflect metabolic deregulation, thus providing insights into system-wide changes in response to physiological challenges or disease processes [1]. Here, a comprehensive urinary metabonomics study on CUMS treated rats was explored using an integration of ^1^H NMR and UPLC-Q-TOF/MS. A total of thirty-six potential biomarkers associated with depression were identified by multivariate statistical analysis of ^1^H NMR and UPLC-Q-TOF/MS data. Among them, twenty potential biomarkers (**1**–**20**) were detected by ^1^H NMR and sixteen (**21**–**36**) were detected by UPLC-Q-TOF/MS.

Interestingly, none of the identified biomarkers were simultaneously detected by both NMR and LC-MS. NMR-based metabonomics studies provide important advantages in untargeted metabolite profile, including robustness, high identification power, superior repeatability and reproducibility [41]. In addition, it is non-discriminating (any compound with protons, carbon, nitrogen or oxygen can be detected) and does not destroy the sample during analysis. Furthermore, NMR may detect compounds that are not retained on an LC column or that are not ionizable in MS and LC-MS. Compared with those advantages of NMR, the destruction of sample, which can cause metabolite losses, and matrix effects (ionization suppression) on coeluting metabolites are problems for LC-MS-based system. In present study, polar metabolites detected by NMR such as pyruvate (**8**) and glucose (**13**) were difficult to detect using LC-MS. Because of their polymeric nature, they cannot be ionized by mass spectrometry, whereas their qualification by NMR is relatively simple [42]. In addition, due to the urine is a complex biological matrix, many metabolites such as amino acids (**1**–**3**, **5**, **7**, **9**, **12**, **15**, **18**) detected by NMR in present study might suffer severe matrix effects by mass spectrometry which didn’t appear in the magnetic detection. However, the inherent limitation of NMR is its poor sensitivity [43]. By contrast, mass spectrometry-based metabonomics offers quantitative analyses with high selectivity and sensitivity, which allow detecting low molecular weight compounds at concentrations lower than the nanogram per milliliter range. Thus, important metabolites present at low concentrations are routinely missed with the NMR-based approach. And, due to the LC-MS allows detection optimized for every compound in a complex mixture, it has better separation effect of complex systems [42]. Here, the derivatives of amino acids (**24**, **30**, **31**, and **34**) were easily detected by LC-MS but missed in NMR detection. The main reason is quite likely their lower concentrations in the urine sample. In addition, metabolites (**21**–**23**, **25**, **27**, and **32**) identified by UPLC-Q-TOF/MS, featured with the large aromatic hydrogen spectra were difficult to be identified by NMR due to the overlap of the peaks with the large aromatic region signals in low-field. Thus, none of the currently available analytical platforms are able to detect the complete range of the metabolites. Our results indicated that the integration of multiple analytical platforms would make up for the deficiencies in different technologies and provide greater scientific power to metabolic disturbances.

In the identified potential biomarkers, to the best of our knowledge, nineteen (**10, 11,** **16, 17, 21–25,** and **27–36**) were reported for the first time as the potential biomarkers of depression. Eight (**1**, **2**, **6**, **12**–**14** and **26**) were firstly identified as potential biomarkers in the urine samples of CUMS-treated rats (Table S1).

The comprehensive metabolic network perturbed by CUMS induced depression was mapped by the integration of ^1^H NMR and UPLC-Q-TOF/MS based metabonomics. Perturbation in CUMS-induced depression involved in twenty-nine metabolic pathways, suggesting depression is a type of complex psychiatric disorder caused by impairment in many different metabolic pathways. Based on the impact value of pathway calculated from pathway topology analysis, the disturbance of valine, leucine and isoleucine biosynthesis; phenylalanine, tyrosine and tryptophan biosynthesis; tryptophan metabolism; and synthesis and degradation of ketone bodies, might play key roles in the onset of depression.

In addition, isoleucine (**1**), leucine (**2**), acetoacetate (**4**), valine (**5**), 3-hydroxybutyric acid (**6**), phenylalanine (**15**), tyrosine (**18**), kynurenic acid (**20**), L-kynurenine (**21**), 5-methoxytryptamine (**22**), indole-3-ehanol (**23**), 3-hydroxykynurenine (**27**), indole-3-acetaldehyde (**32**), 2-aminomuconic acid semialdehyde (**35**) and 2-amino-3-carboxymuconic acid semialdehyde (**36**) involved in the above metabolic pathways which make a greater contribution to the onset of depression may denote their potential as targeted biomarkers for differentiating CUMS and normal states.

Notably, there are eight potential biomarkers (**20**–**23**, **27**, **32**, **35**, and **36**) involved in the tryptophan metabolism. Tryptophan (TRP) is the precursor of serotonin or 5-hydroxytryptamine (5-HT) and L-kynurenine, two neuromodulators that are critically implicated in the regulation of depression. Although the levels of TRP and 5-HT did not change in our study, some of the products generated by the 5-HT metabolic and kynurenine pathways were detected. These findings aroused our interest in exploring the expression of related proteins. Two key enzymes involved in tryptophan metabolism, DDC (EC 4.1.1.28) and IDO (EC: 1.13.11.52) were assayed by Western blotting (Figure 4A and B). Up-regulation of DDC and down-regulation of IDO were observed in the hippocampus of CUMS-treated rats, in accordance with the results of our metabonomics approach. The excessive synthesis of 5-MT required an increased amount of 5-HT; thus, this requirement could have activated DDC to metabolize 5-HTP into 5-HT. However, we did not observe increased level of 5-HT. On the contrary, the levels of kynurenine and its metabolites in the kynurenine pathway of tryptophan metabolism were decreased significantly, and inhibited expression of IDO was also detected. Thus, we speculated that DDC was activated to degrade 5-HTP into 5-HT for the synthesis of 5-MT, which resulted in the depletion of tryptophan. Therefore, IDO expression was inhibited, leading to decreased kynurenine production. Not only depletion of tryptophan and, consequently, 5-HT but also production of 5-MT, induction of aromatic L-amino acid decarboxylase (DDC) and inhibition of indoleamine 2, 3-dioxygenase (IDO) are involved in the pathophysiology of depression. More evidence is necessary to confirm whether these factors are associated with the onset and the underlying molecular mechanisms of depression.

### Conclusions

An integrated approach utilizing ^1^H NMR and UPLC-Q-TOF/MS was firstly applied for a comprehensive urinary metabonomics study on the CUMS-treated rats. Thirty-six potential biomarkers were identified by the two different analytical techniques. Among the identified potential biomarkers, nineteen (**10, 11,** **16, 17, 21–25,** and **27–36**) were firstly reported as potential biomarkers of CUMS-induced depression. Perturbation in CUMS-induced depression involved in twenty-nine metabolic pathways, suggesting depression is a type of complex psychiatric disorder caused by impairment in many different metabolic pathways. Consequently, this paper presented a comprehensive map of the metabolic pathways perturbed by CUMS and expanded on the multitude of potential biomarkers that has been previously reported in the CUMS model. Valine, leucine and isoleucine biosynthesis; phenylalanine, tyrosine and tryptophan biosynthesis; tryptophan metabolism; and the synthesis and degradation of ketone bodies would be considered as the most influenced metabolic pathways associated with CUMS-induced depression. Isoleucine (**1**), leucine (**2**), acetoacetate (**4**), valine (**5**), 3-hydroxybutyric acid (**6**), phenylalanine (**15**), tyrosine (**18**), kynurenic acid (**20**), L-kynurenine (**21**), 5-methoxytryptamine (**22**), indole-3-ehanol (**23**), 3-hydroxykynurenine (**27**), indole-3-acetaldehyde (**32**), 2-aminomuconic acid semialdehyde (**35**) and 2-amino-3-carboxymuconic acid semialdehyde (**36**) involved in the above four metabolic pathways may denote their potential as targeted biomarkers for differentiating CUMS and normal states. Monitoring changes of these metabolites may predict the development of depression. Moreover, the results of Western blot analysis of DDC and IDO in the hippocampus of CUMS-treated rats indicated that depletion of 5-HT and tryptophan, production of 5-MT and altered expression of DDC and IDO together played a key role in the initiation and progression of depression. In addition, among the identified potential biomarkers, twenty (**1**–**20**) were detected by ^1^H NMR and sixteen (**21**–**36**) were detected by UPLC-Q-TOF/MS. None of the potential biomarkers were detected by NMR and LC-MS simultaneously. Therefore, the integration of ^1^H NMR and UPLC-Q-TOF/MS in metabonomics study will provide a comprehensive approach to identify the most full-scale metabolome coverage and a more in-depth understanding of the pathophysiological processes of disease.

## Supporting Information

Figure S1 **The flow chart of the metabonomics study based on integrated ^1^H NMR and UPLC-Q-TOF/MS techniques for the urinary metabolic profiles of CUMS-induced depression.**
(TIF)

Figure S2 **PCA score plots of naïve and CUMS-treated rats.**
(TIF)

Figure S3 **Ethological changes in the CUMS-treated rats.**
(TIF)

Figure S4 **Typical ^1^H NMR spectra of urine samples from naïve and CUMS-treated rats.**
(TIF)

Figure S5 **The UPLC-Q-TOF/MS base peak chromatograms of urine samples in the positive and negative modes, respectively.**
(TIF)

Figure S6 **Summary of pathway analysis with MetPA.**
(TIF)

Table S1 **The potential biomarkers related to CUMS-treated rats in the previous literatures.**
(DOCX)

Table S2 **The reproducibility and precision of UPLC-Q-TOF/MS method validation under the positive and negative ion modes using QC sample.**
(DOCX)

Table S3 **Result from ingenuity analysis with MetPA.**
(DOCX)
