# Supplementary figures and images for: Integration of 1H NMR and UPLC-Q-TOF/MS for a Comprehensive Urinary Metabonomics Study on a Rat Model of Depression Induced by Chronic Unpredictable Mild Stress
Source: PLoS One. 2013 May 17;8(5):e63624. doi: 10.1371/journal.pone.0063624 (PMC3656962; doi:10.1371/journal.pone.0063624)

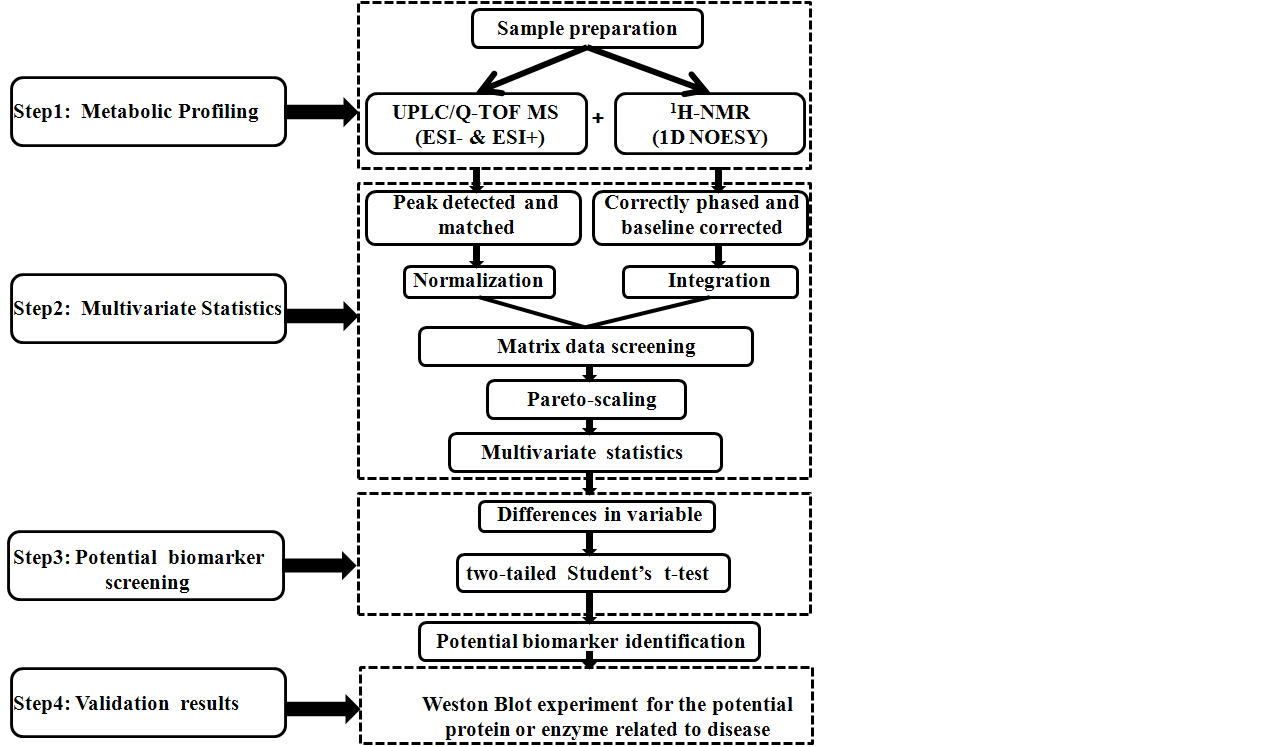

Supplement: Figure S1 — The flow chart of the metabonomics study based on integrated 1H NMR and UPLC-Q-TOF/MS techniques for the urinary metabolic profiles of CUMS-induced depression. (TIF) [file pone.0063624.s001.tif]

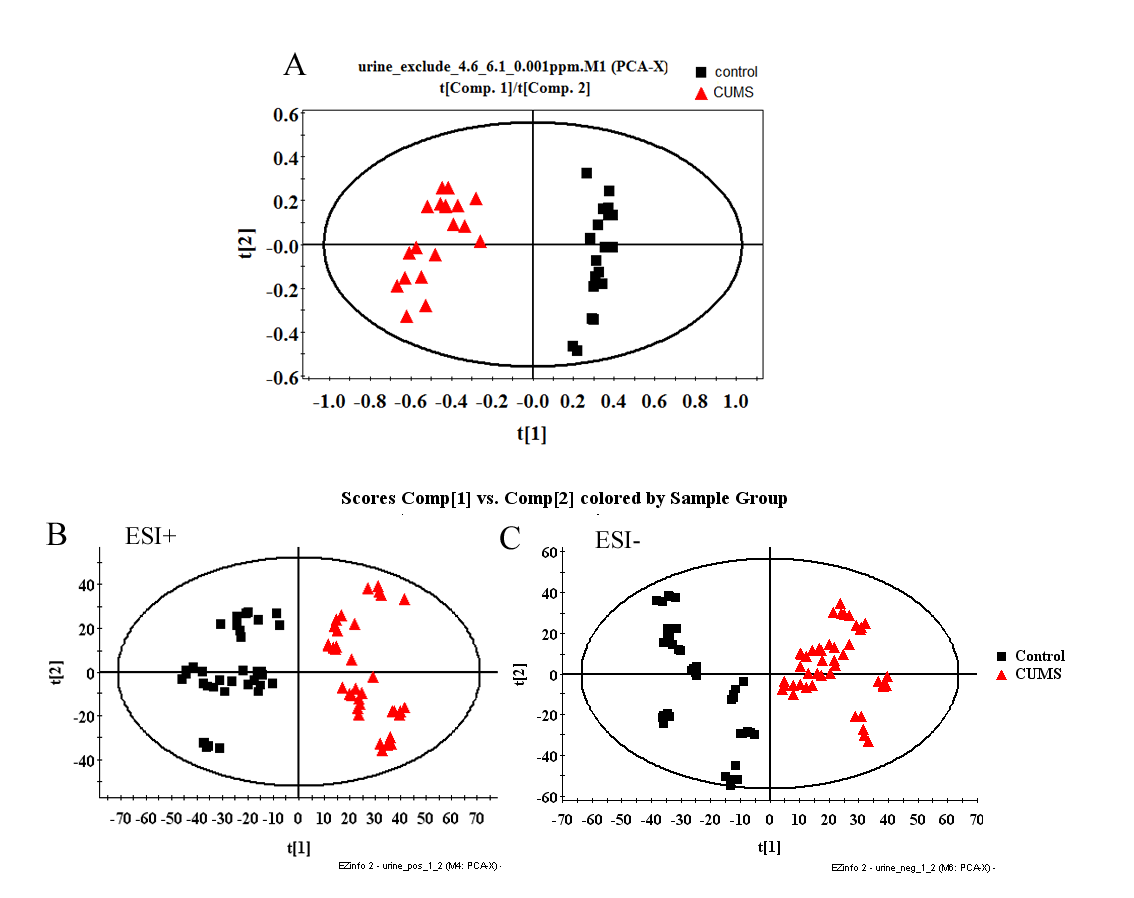

Supplement: Figure S2 — PCA score plots of naïve and CUMS-treated rats. (TIF) [file pone.0063624.s002.tif]

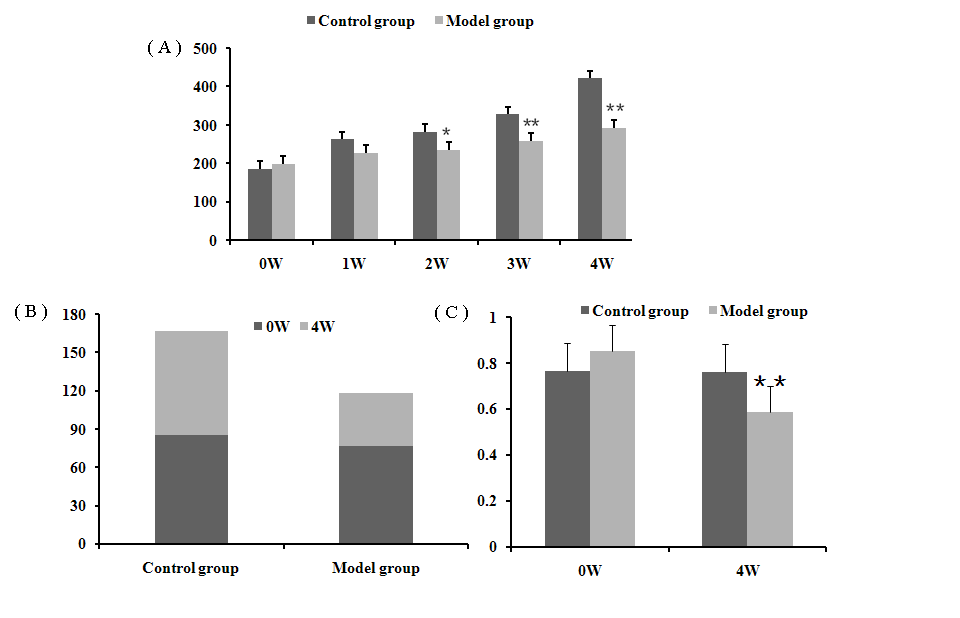

Supplement: Figure S3 — Ethological changes in the CUMS-treated rats. (TIF) [file pone.0063624.s003.tif]

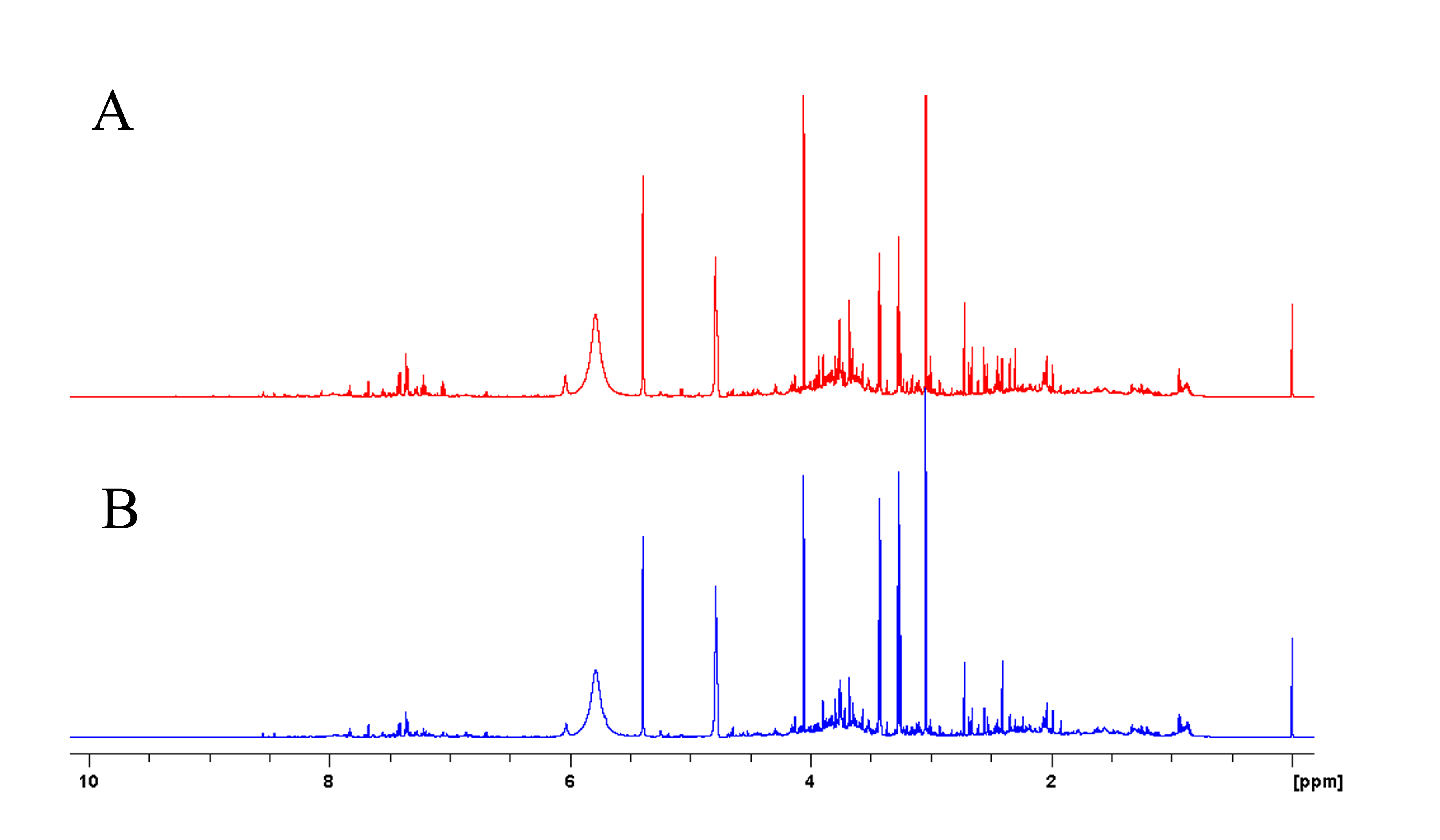

Supplement: Figure S4 — Typical 1H NMR spectra of urine samples from naïve and CUMS-treated rats. (TIF) [file pone.0063624.s004.tif]

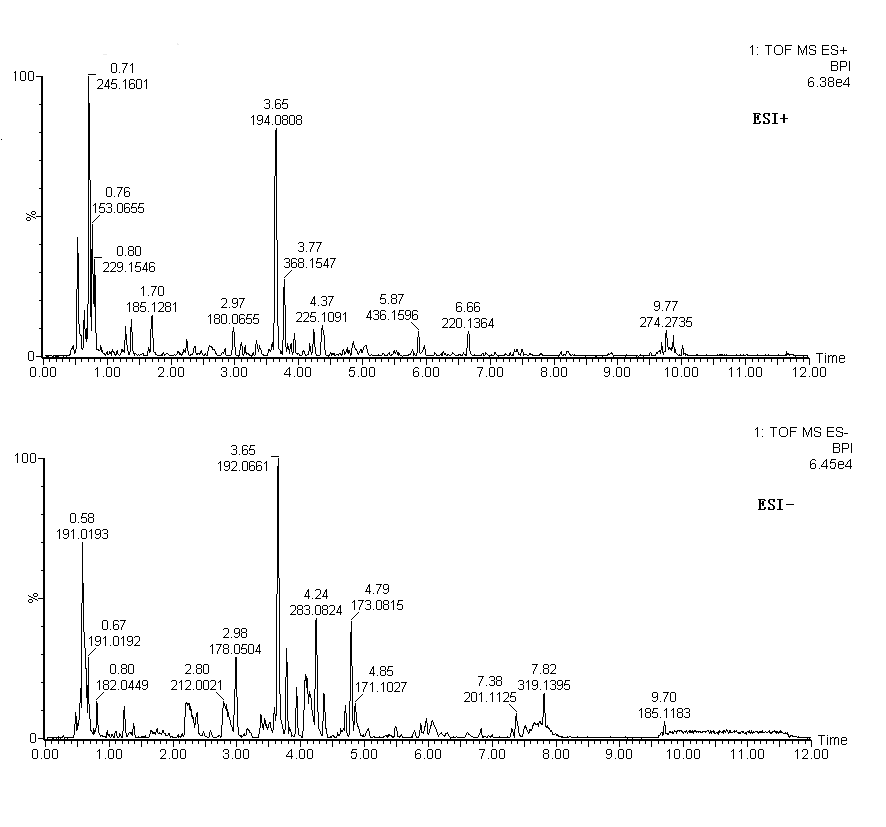

Supplement: Figure S5 — The UPLC-Q-TOF/MS base peak chromatograms of urine samples in the positive and negative modes, respectively. (TIF) [file pone.0063624.s005.tif]

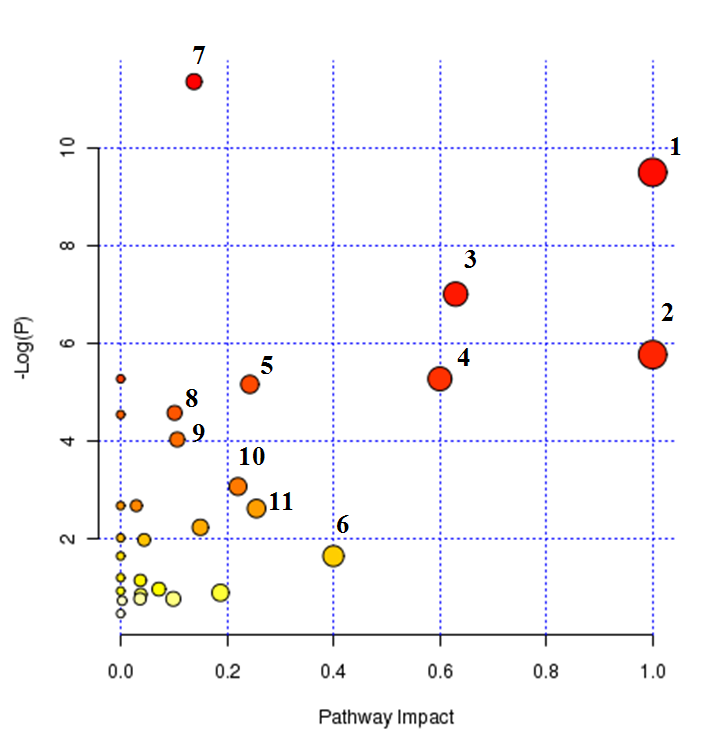

Supplement: Figure S6 — Summary of pathway analysis with MetPA. (TIF) [file pone.0063624.s006.tif]
