# Supplementary material for: Integration of 1H NMR and UPLC-Q-TOF/MS for a Comprehensive Urinary Metabonomics Study on a Rat Model of Depression Induced by Chronic Unpredictable Mild Stress
Source: PLoS One. 2013 May 17;8(5):e63624. doi: 10.1371/journal.pone.0063624 (PMC3656962; doi:10.1371/journal.pone.0063624)
Supplement: Table S1 — The potential biomarkers related to CUMS-treated rats in the previous literatures. (DOCX) [file pone.0063624.s007.docx]

Table S1. The potential biomarkers related to CUMS-treated rats in the previous literatures.

| **Sample** | **Biomarker** | **Detection technique** | | **Authors** | | **Published Year** | | **Journal** |
| --- | --- | --- | --- | --- | --- | --- | --- | --- |
| **Urine** |  | **GC-MS** | | **Wang, *et al.*** | | **2009,8, 2511–2518.** | | **Journal of Proteome Research** |
|  | Homovanillate; Glutamine ; Pimelate ; Proline; Citrate ; Glutamate; Hippurate; tyramine; Hexadecanoate; Methionine; Threonine ; Succinate ; Hexanedioate ; phenylalanine ; Valine ; Suberate ; Tyrosine; 5-Hydroxyindoleacetate ; Tryptophan ; 4-aminohippurate | | | | | | | |
| **Urine** |  | **UPLC-MS** | | **Zheng, *et al.*** | | | **(2010);411;204–209** | **Clinica Chimica Acta** |
|  | Phenylalanine ; Tryptophan ; creatinine ; Kynurenic acid ; Phenylacetylglycine ; Indole-3-acetate  Hippurate; Xanthurenic acid ; N2-succinyl-L-ornithine ; Citrate ; Ketoglutarate ; Indoxyl sulfate | | | | | | | |
| **Urine** |  | **GC-MS** | | **Dai, *et al.*** | | | **2010 (128) 482–489** | **Journal of Ethnopharmacology** |
|  | Hippuric acid; Glycine ; Phenylpyruvic acid ; Osalic acid ; Hexadecanoic acid ; 8,10-octadecadienoic acid; 2-furancarboxylic acid ; Hexanedioic acid ; Tyrosine | | | | | | | |
| **Urine** |  | **UPLC-MS** | | **Su, *et al.*** | | | **2011 (55) 533–539** | **Journal of Pharmaceutical and Biomedical Analysis** |
|  | Pantothenic acid; 3-O-methyldopa ; Kynurenic acid ; Xanhthurenic acid ; 2,8-dihydroxyquinoline glucuronide ; 5-hydroxy-6-methoxyindole glucurnoide ; L-phenylalanyl-L-hydroxyproline ; Indole-3-carboxylic acid ; proline | | | | | | | |
| **Urine** |  | **NMR** | | **Zhou,*et al.*** | | | **2011 (137) 236– 244** | **Journal of Ethnopharmacology** |
|  | Creatine ; Lactate ; Taurine ; Pyruvate ; Phenylacetylglycine; Betaine | | | | | | | |
| **Urine** |  | **NMR** | | **Liu, *et al.*** | | | **2012, 50, 187–192** | **Magn. Reson. Chem.** |
|  | DMG ; Glycine; 2-OG ; Citrate ; Succinate ; Acetate ; Pyruvate ; Glutamine ; asparagine | | | | | | | |
| **Urine** |  | **UPLC-MS** | | **Gao, *et al.*** | | | **(2012)** | **Phytother. Res.** |
|  | Xanthurenic acid ; Creatinine ; Phenylacetylglycine ; 7-methylxanthine ; Xanthosine ; Tyrosine ; Phenylalanine; Kynurenic acid ; Hippurate ; Tryptophan ; N2-succinyl-L-ornithine ; Indole-3-acetate; Phenylacetylglycine; Indoxyl sulfate ; Ketoglutarate ; 3-hydroxyphenyl propionic acid sulfate ; citrate ; Succinic acid | | | | | | | |
| **Plasma** |  | | **GC-MS** | | **Li, *et al.*** | | **2010;24: 3539–3546** | **Rapid Commun. Mass Spectrom.** |
|  | Glutamic acid ; Tryptophan ; Hexadecanoic acid ; Tyrosine ; Linoleate ; Octadecanoic acid ; Glycine  Butanedioic acid ; 2,3-dihydroxypropanoic acid ; Glutamine ; Fructose ; glucose | | | | | | | |
| **Plasma** |  | | **NMR and UPLC-MS** | | **Zheng,*et al.*** | | **2010** | **Metabolomics** |
|  | (**NMR**): N-acetyl glycoproteins; Lactate; Acetoacetate; 3-hydroxybutyrate; Glutamine ; Valine; Choline; glucose  **(UPLC-MS**): Tryptophan; 3-indolepropionic acid; Phenylalanine; Deoxycytidine; C20:4 lysophosphatidylcholine; C14:0 lysophosphatidylcholine; C16:0 lysophosphatidylcholine; Uric acid; Cholic acid; C22:6 lysophosphatidylcholine | | | | | | | |
| **Plasma** |  | | **GC-MS** | | **Gao, *et al.*** | | **2011 (137) 690– 699** | **Journal of Ethnopharmacology** |
|  | Butanedioic acid; 2,3-dihydroxy-propanoic acid; Glycine ; glutamine ; Glutamic acid; Fructose ; Glucose ; Tyrosine; Hexadecanoic acid; Tryptophan; Linoleate; Octadecanoic acid | | | | | | | |
| **Plasma** |  | | **NMR** | | **Liu, *et al.*** | | **2012; 64, 578–588** | **Royal Pharmaceutical Society *Journal of Pharmacy and Pharmacology*,** |
|  | Glycoprotein; Choline; Lactate ; *β*-hydroxybutyrate ; Alanine ; Trimethylamine-N-oxide(TMAO) ; Valine ; Leucine/isoleucine | | | | | | | |
| **Brain** |  | | **GC-MS** | | **Ni, *et al.*** | | **2008 (582) 2627–2636** | **FEBS Letters** |
|  | Imidazole-4-acetate ; Lactate ; Methylimidazole acetate ; Alanine ; Glycine ; Valine ; serine ; Leucine ; Iso-leucine ; Threonine ; Proline ; Asparagines ; N-acetyl aspartate ; 3-indolepropionate ; Aspartate ; Methionine ; Glutamate ; Glutamine ; Phenylalanine ; Cysteine ; Hexadecanoic acid ; Lysine ; Arachidonic acid ; tryptophan | | | | | | | |
| **Heart sample** |  | | **GC-MS** | | **Zhang,*et al.*** | | **2012 (70) 534– 538** | **Journal of Pharmaceutical and Biomedical Analysis** |
|  | Inosine ; Glucose ; 9,12-octadecadienoic acid ; Hexadecanoic acid ; Glutamine ; Octadecanoic acid ; Proline ; Urea ; Creatinine ; asparagines | | | | | | | |
