# Supplementary material for: Integration of 1H NMR and UPLC-Q-TOF/MS for a Comprehensive Urinary Metabonomics Study on a Rat Model of Depression Induced by Chronic Unpredictable Mild Stress
Source: PLoS One. 2013 May 17;8(5):e63624. doi: 10.1371/journal.pone.0063624 (PMC3656962; doi:10.1371/journal.pone.0063624)
Supplement: Table S2 — The reproducibility and precision of UPLC-Q-TOF/MS method validation under the positive and negative ion modes using QC sample. (DOCX) [file pone.0063624.s008.docx]

**Table S2.** The reproducibility and precision of UPLC-Q-TOF/MS method validation under the positive and negative ion modes using QC sample.

Reproducibility of UPLC-Q-TOF/MS method

| **Positive** | | | | | | | **Negative** | | | | | | |
| --- | --- | --- | --- | --- | --- | --- | --- | --- | --- | --- | --- | --- | --- |
| **NO** | **RT** | **SD** | **RSD%** | ***m/z*** | **SD** | **RSD%** | **NO** | **RT** | **SD** | **RSD%** | ***m/z*** | **SD** | **RSD%** |
| 1 | 1.31 | 0 | 0 | 98.0603 | 0.0004 | 0.0004 | 1 | 0.80 | 0.0042 | 0.3453 | 182.0459 | 0.0003 | 0.0002 |
| 2 | 1.47 | 0.0071 | 0.4818 | 297.1443 | 0.0007 | 0.0002 | 2 | 1.22 | 0.0067 | 0.3115 | 144.0670 | 0.0005 | 0.0001 |
| 3 | 1.73 | 0.0035 | 0.2042 | 126.0919 | 0.0005 | 0.0004 | 3 | 2.37 | 0.0045 | 0.3124 | 158.0817 | 0.0006 | 0.0003 |
| 4 | 3.01 | 0.0053 | 0.1775 | 105.0334 | 0.0004 | 0.0004 | 4 | 2.98 | 0.0046 | 0.1546 | 178.0510 | 0.0003 | 0.0002 |
| 5 | 3.17 | 0.0053 | 0.1776 | 372.2382 | 0.0012 | 0.0003 | 5 | 3.64 | 0.0034 | 0.2137 | 192.0669 | 0.0009 | 0.0004 |
| 6 | 3.68 | 0.0053 | 0.1684 | 91.0541 | 0.0002 | 0.0002 | 6 | 3.79 | 0.0037 | 0.2107 | 349.1133 | 0.0003 | 0.0003 |
| 7 | 4.43 | 0.0064 | 0.1742 | 130.0654 | 0.0005 | 0.0004 | 7 | 4.25 | 0.0052 | 0.1631 | 283.0823 | 0.0004 | 0.0002 |
| 8 | 5.61 | 0.0053 | 0.1206 | 170.0612 | 0.0010 | 0.0006 | 8 | 4.77 | 0.0031 | 0.1206 | 173.0825 | 0.0006 | 0.0004 |
| 9 | 5.93 | 0.0046 | 0.0780 | 243.1021 | 0.0008 | 0.0003 | 9 | 5.48 | 0.0071 | 0.0461 | 197.0815 | 0.0005 | 0.0003 |
| 10 | 6.68 | 0.0035 | 0.0529 | 203.1108 | 0.0006 | 0.0003 | 10 | 7.36 | 0.0039 | 0.0438 | 201.1137 | 0.0007 | 0.0002 |

Precision of UPLC-Q-TOF/MS method

| **Positive** | | | | | | | **Negative** | | | | | | |
| --- | --- | --- | --- | --- | --- | --- | --- | --- | --- | --- | --- | --- | --- |
| **NO** | **RT** | **SD** | **RSD%** | ***m/z*** | **SD** | **RSD%** | **NO** | **RT** | **SD** | **RSD%** | ***m/z*** | **SD** | **RSD%** |
| 1 | 1.31 | 0 | 0 | 98.0601 | 0.0002 | 0.0002 | 1 | 0.80 | 0.0039 | 0.3127 | 182.0459 | 0.0004 | 0.0003 |
| 2 | 1.47 | 0.0055 | 0.3739 | 297.1446 | 0.0008 | 0.0003 | 2 | 1.22 | 0.0056 | 0.2945 | 144.0670 | 0.0006 | 0.0003 |
| 3 | 1.73 | 0.0041 | 0.2362 | 126.0916 | 0.0005 | 0.0004 | 3 | 2.37 | 0.0034 | 0.3067 | 158.0817 | 0.0007 | 0.0004 |
| 4 | 3.01 | 0.0041 | 0.1357 | 105.0334 | 0.0005 | 0.0005 | 4 | 2.98 | 0.0042 | 0.1476 | 178.0510 | 0.0004 | 0.0002 |
| 5 | 3.17 | 0.0041 | 0.1287 | 372.2387 | 0.0014 | 0.0004 | 5 | 3.64 | 0.0040 | 0.1978 | 192.0669 | 0.0005 | 0.0003 |
| 6 | 3.68 | 0.0082 | 0.2221 | 91.0540 | 0.0005 | 0.0005 | 6 | 3.79 | 0.0041 | 0.2091 | 349.1133 | 0.0004 | 0.0002 |
| 7 | 4.43 | 0.0063 | 0.1428 | 130.0652 | 0.0005 | 0.0004 | 7 | 4.25 | 0.0045 | 0.1856 | 283.0823 | 0.0003 | 0.0003 |
| 8 | 5.61 | 0.0041 | 0.0728 | 170.0609 | 0.0011 | 0.0007 | 8 | 4.77 | 0.0027 | 0.1371 | 173.0825 | 0.0005 | 0.0002 |
| 9 | 5.93 | 0 | 0 | 243.1017 | 0.0014 | 0.0006 | 9 | 5.48 | 0.0053 | 0.0375 | 197.0815 | 0.0004 | 0.0002 |
| 10 | 6.68 | 0.0052 | 0.0773 | 203.1108 | 0.0006 | 0.0003 | 10 | 7.36 | 0.0031 | 0.0398 | 201.1137 | 0.0006 | 0.0004 |
