# Supplementary material for: Integration of 1H NMR and UPLC-Q-TOF/MS for a Comprehensive Urinary Metabonomics Study on a Rat Model of Depression Induced by Chronic Unpredictable Mild Stress
Source: PLoS One. 2013 May 17;8(5):e63624. doi: 10.1371/journal.pone.0063624 (PMC3656962; doi:10.1371/journal.pone.0063624)
Supplement: Table S3 — Result from ingenuity analysis with MetPA. (DOCX) [file pone.0063624.s009.docx]

Table S3. Result from ingenuity analysis with MetPA.

| NO. |  | Total | Expected | Hits | Raw p | Impact |
| --- | --- | --- | --- | --- | --- | --- |
| 1 | Valine, leucine and isoleucine biosynthesis | 11 | 0.26 | 4 | 7.49E-05 | 1 |
| 2 | Phenylalanine, tyrosine and tryptophan biosynthesis | 4 | 0.09 | 2 | 3.13E-03 | 1 |
| 3 | Tryptophan metabolism | 41 | 0.97 | 8 | 9.07E-04 | 0.63 |
| 4 | Synthesis and degradation of ketone bodies | 5 | 0.12 | 2 | 5.14E-03 | 0.6 |
| 5 | Methane metabolism | 9 | 0.21 | 1 | 1.93E-01 | 0.4 |
| 6 | Tyrosine metabolism | 42 | 0.99 | 3 | 7.31E-02 | 0.26 |
| 7 | Glycine, serine and threonine metabolism | 32 | 0.75 | 4 | 5.75E-03 | 0.24 |
| 8 | Histidine metabolism | 15 | 0.35 | 2 | 4.66E-02 | 0.22 |
| 9 | Pyruvate metabolism | 22 | 0.52 | 1 | 4.10E-01 | 0.19 |
| 10 | Alanine,aspartate and glutamate metabolism | 24 | 0.56 | 2 | 1.07E-01 | 0.15 |
| 11 | Aminoacyl-tRNA biosynthesis | 67 | 1.58 | 9 | 1.17E-05 | 0.14 |
| 12 | Arginine and proline metabolism | 44 | 1.04 | 4 | 1.78E-02 | 0.11 |
| 13 | Butanoate metabolism | 20 | 0.47 | 3 | 1.03E-02 | 0.1 |
| 14 | Glycolysis or Gluconeogenesis | 26 | 0.61 | 1 | 4.65E-01 | 0.1 |
| 15 | Citrate cycle (TCA cycle) | 20 | 0.47 | 1 | 3.81E-01 | 0.07 |
| 16 | Cysteine and methionine metabolism | 28 | 0.66 | 2 | 1.39E-01 | 0.04 |
| 17 | Glyoxylate and dicarboxylate metabolism | 16 | 0.38 | 1 | 3.18E-01 | 0.04 |
| 18 | Starch and sucrose metabolism | 23 | 0.54 | 1 | 4.24E-01 | 0.04 |
| 19 | Galactose metabolism | 26 | 0.61 | 1 | 4.65E-01 | 0.04 |
| 20 | Phenylalanine metabolism | 9 | 0.21 | 3 | 6.90E-02 | 0.03 |
| 21 | D-Glutamine and D-glutamate metabolism | 5 | 0.12 | 2 | 5.14E-03 | 0 |
| 22 | Valine, leucine and isoleucine degradation | 38 | 0.89 | 4 | 1.07E-02 | 0 |
| 23 | Ubiquinone and other terpenoid-quinone biosynthesis | 3 | 0.07 | 1 | 6.90E-02 | 0 |
| 24 | Cyanoamino acid metabolism | 6 | 0.14 | 1 | 1.33E-01 | 0 |
| 25 | Nitrogen metabolism | 9 | 0.21 | 1 | 1.93E-01 | 0 |
| 26 | Pantothenate and CoA biosynthesis | 15 | 0.35 | 1 | 3.02E-01 | 0 |
| 27 | Sphingolipid metabolism | 21 | 0.49 | 1 | 3.96E-01 | 0 |
| 28 | Purine metabolism | 68 | 1.6 | 2 | 4.82E-01 | 0 |
| 29 | Pyrimidine metabolism | 41 | 0.97 | 1 | 6.29E-01 | 0 |

Note: Total is the total number of compounds in the pathway; the Hits is the actually matched number from the user uploaded data; the Raw p is the original p value calculated from the enrichment analysis; the Impact is the pathway impact value calculated from pathway topology analysis.
